# Unexpectedly high rate of unrecognized acute kidney injury and its trend over the past 14 years

**DOI:** 10.1038/s41598-025-88732-8

**Published:** 2025-02-21

**Authors:** Lina Han, Hongxiao Li, Lingfan Luo, Xiaolan Ye, Yan Ren, Zimeng Xu, Wei Zhang, Jiawei Zhang, Yiwen Li, Bin Chen, Bin Zhu, Lina Shao

**Affiliations:** 1 Kidney Department, Zhenhai People’s Hospital (Ningbo No.7 Hospital), 718 Nanerxi Road, Luotuo Subdistrict, Zhenhai, Ningbo, China; 2 Urology and Nephrology Center, Department of Nephrology, Zhejiang Provincial People’s Hospital (Affiliated People’s Hospital), Hangzhou Medical College, Hangzhou, Zhejiang China; 3 Division of Health Sciences, Hangzhou Normal University, Hangzhou, China; 4 Center for Clinical Pharmacy, Department of Pharmacy, Hangzhou Medical College, Zhejiang Provincial People’s Hospital (Affiliated People’s Hospital), Cancer Center, Hangzhou, Zhejiang China

**Keywords:** Acute kidney injury, Unrecognized rate, Risk factors, Survival prognosis, Acute kidney injury, Epidemiology

## Abstract

Acute kidney injury (AKI) is a frequent yet often overlooked complication. This study examines the incidence, unrecognized rate, and outcomes of AKI in adults at a large public Chinese hospital from 2010 to 2023. AKI rates were calculated, and outcomes were assessed using follow-up records. Multivariate logistic regression identified risk factors for unrecognized AKI. Among 2,790,540 patients, 5,080 met the AKI criteria, with an overall incidence of 0.18% (0.78% in hospitalizations, 0.05% in outpatients). The unrecognized AKI was 76.3%. In this group, 75% were stage 1, 16.7% stage 2, and 8.3% stage 3. Orthopedics had the highest unrecognized rate (94.5%) and ICUs the lowest (55.77%). Unrecognition of AKI improved from 90.3% in 2010–2011 to 70.2% in 2022–2023. AKI stage progression was linked to poorer survival. Patients with recognized AKI recovered faster than those with unrecognized AKI (8.0 vs. 9.0 days, p < 0.001). The mean follow-up time was 15.8 days, with similar rates at 28 and 90 days post-AKI for both groups. Risk factors for unrecognized AKI included lower AKI stage, baseline creatinine, absence of shock/heart disease/hypertension, and non-nephrology/surgery admissions. Non-nephrology physicians’ unfamiliarity with AKI guidelines may contribute to low awareness. Improved early detection and monitoring in high-risk groups are needed.

**Supplementary Information:**

The online version contains supplementary material available at 10.1038/s41598-025-88732-8.

## Introduction

Acute kidney injury (AKI) is a complex syndrome caused by several etiologies and is characterized by a sudden decrease in kidney function^1^. Its onset and development are marked by a complicated state of illness that is difficult to treat. Early diagnosis of AKI is challenging, and the condition is easily unrecognized, leading to a high mortality rate^2,3^. A nationwide cross-sectional survey conducted ten years ago in China reported an incidence of AKI of 7.2% among 2,223,230 hospitalized adults. However, the unrecognized rate of AKI was 74.2%^4^, suggesting that these patients were not receiving appropriate renal care. Previous research has shown that early recognition and treatment of AKI can significantly improve patient outcomes, reducing both mortality and the incidence of complications^5,6^.

Furthermore, the inadequate long-term follow-up for AKI patients post-discharge is a critical issue impacting their prognosis. Despite recent advances in AKI management, follow-up rates after discharge remain low, leading to a lack of long-term monitoring and management of patients with AKI. Patients with severe AKI who received inpatient renal replacement therapy and were evaluated by the nephrology department within 90 days after discharge had a significantly lower two-year mortality rate than those who did not receive nephrology follow-up^7^. Only 26% of patients with AKI receive follow-up care within six months after discharge^8^. High rates of unrecognized AKI and low rates of follow-up lead to increased mortality among patients with AKI, increased governmental healthcare expenditure, and several other issues^9^.

To better manage patients with AKI, it is important to fully understand the prevalence, missed diagnosis rate, and follow-up rate of AKI in local areas. Although an epidemiological survey of AKI in Chinese hospitals was published ten years ago^4^, there is an urgent need to understand the incidence, missed diagnosis, and follow-up rates of AKI in real clinical practice since the publication of the definition and clinical practice guidelines of AKI by Kidney Disease Improving Global Outcomes (KDIGO) in 2012^1^.

This retrospective cohort study used existing electronic medical record data, allowing for the analysis of large sample sizes over extended periods and providing a wealth of clinical information. The primary objective of this study was to evaluate the recognition rates of AKI and the impact on patient prognosis in a large public hospital. The study aimed to provide a detailed assessment of the current state of AKI in China.

## Methods

### Study design and setting

This retrospective cohort study was conducted using electronic medical record data from a medical big data platform at Zhejiang Provincial People’s Hospital. The Institutional Ethics Committee approved this study (approval number: QT2024011). Informed consent was waived from all subjects because of the retrospective study. All methods were performed in accordance with the relevant guidelines and regulations.

### Selection of participants

Patients > 18 years of age who had more than two serum creatinine tests within seven days in the hospital’s outpatient department or during hospitalization were included in this study.

Patients with no baseline or follow-up creatinine values or an incomplete medical history were excluded. Those with chronic kidney disease (CKD) stage V, those who required dialysis, and those who had received a renal transplant prior to the index admission were also excluded^10^.

### Definition of baseline creatinine

The definition of baseline creatinine in this study was established based on two scenarios: 1. According to the inclusion criteria of our study, patients admitted to the study were required to have at least two creatinine measurements within seven days of admission. We adopted the AKI definition recommended by KDIGO, which specifies an increase in creatinine of ≥ 26.5 μmol/L (0.3 mg/dL) within 48 h or a rise to more than 1.5 times the baseline value within seven days. If a patient met the AKI criteria, the preceding creatinine measurement was designated as the baseline creatinine value for that patient, thereby including any AKI events occurring during hospitalization. 2. For patients not meeting the AKI criteria, the first creatinine measurement obtained during their hospital stay was defined as the baseline creatinine value.

### Definition of AKI

The 2012 KDIGO definition of AKI was used as the major screening criteria in this study. An increase in the serum creatinine (SCr) level ≥ 0.3 mg/dL within 48 h or ≥ 1.5 times the baseline within seven days was defined as AKI^1^. As records of urine volume were mostly missing from the electronic medical record system and relevant data could not be collected, urinary output data were not used to define AKI in the current study.

### The process for AKI recognition

We employed a hybrid approach, integrating an automated algorithm with manual medical record review, to identify patients with AKI during treatment. We then analyzed the rate of unrecognized AKI cases by comparing our findings with AKI diagnoses recorded in the electronic medical record system. Our hospital’s data platform, provided by Yidu Cloud Technology Company Ltd and integrated with the electronic medical record system, enabled us to automatically identify the target population based on predefined inclusion and exclusion criteria. Two designated authors were responsible for data cleaning and verification of relevant diagnoses, while two additional authors conducted the review and staging of AKI cases.

### Prognostic classification of AKI

The prognostic classification of AKI was based on the extent of the reduction of serum creatinine levels within 90 days after AKI. Full recovery was defined as serum creatinine that returned to baseline levels or lower. Partial recovery was defined as a ≥ 50% decrease from the peak but higher than the baseline blood creatinine level. A < 50% decrease from the peak value or patients dependent on dialysis treatment were classified as unrecovered^10^.

### Data Source

This study was conducted at Zhejiang Provincial People’s Hospital in Zhejiang, China. Electronic medical records of patients attending the hospital from January 1, 2010, to December 31, 2023, were used to identify suitable patients and collect relevant data. The diagnosis of AKI was confirmed using electronic medical records, which also provided details regarding the patients’ general demographic information, kidney function tests, mortality, and follow-up.

### Data collection and processing

Information collected during outpatient visits or hospital admissions included age, sex, serum creatinine level, and inpatient department status. Comorbidities were identified based on the diagnosis codes at admission and discharge. Complications included hypertension, diabetes, coronary heart disease, tumors, and CKD. The follow-up status of patients 28 and 90 days after discharge was determined using all relevant medical records, including those from the Hospital Information System, Laboratory Information System, and outpatient records.

### Statistical analysis

All statistical analyses were conducted using IBM SPSS Statistics (Version 26.0, IBM Corp., Armonk, NY, USA). The Kolmogorov–Smirnov test was used to assess the normality of the variables. Quantitative data are presented as either means with standard deviations or medians with interquartile ranges, and qualitative data are presented as percentages. The t-test was used to compare data with a normal distribution, and the rank-sum test was used to assess data that did not conform to a normal distribution. The chi-square test was used to compare qualitative data. To identify the risk factors for unrecognized AKI, logistic regression analysis was performed for a univariate analysis of unrecognized AKI, and the variables with a p-value < 0.01 in the univariate analysis were used as the candidate variables in the multivariate logistic regression analysis. The independent variables were checked for collinearity using the variance inflation factor (VIF) and tolerance test. Survival differences according to the KDIGO stages of AKI were visualized using Kaplan–Meier survival plots. All statistical tests were two-tailed, and significance was set at p < 0.05.

## Results

### Incidence of AKI

**Fig. 1:**
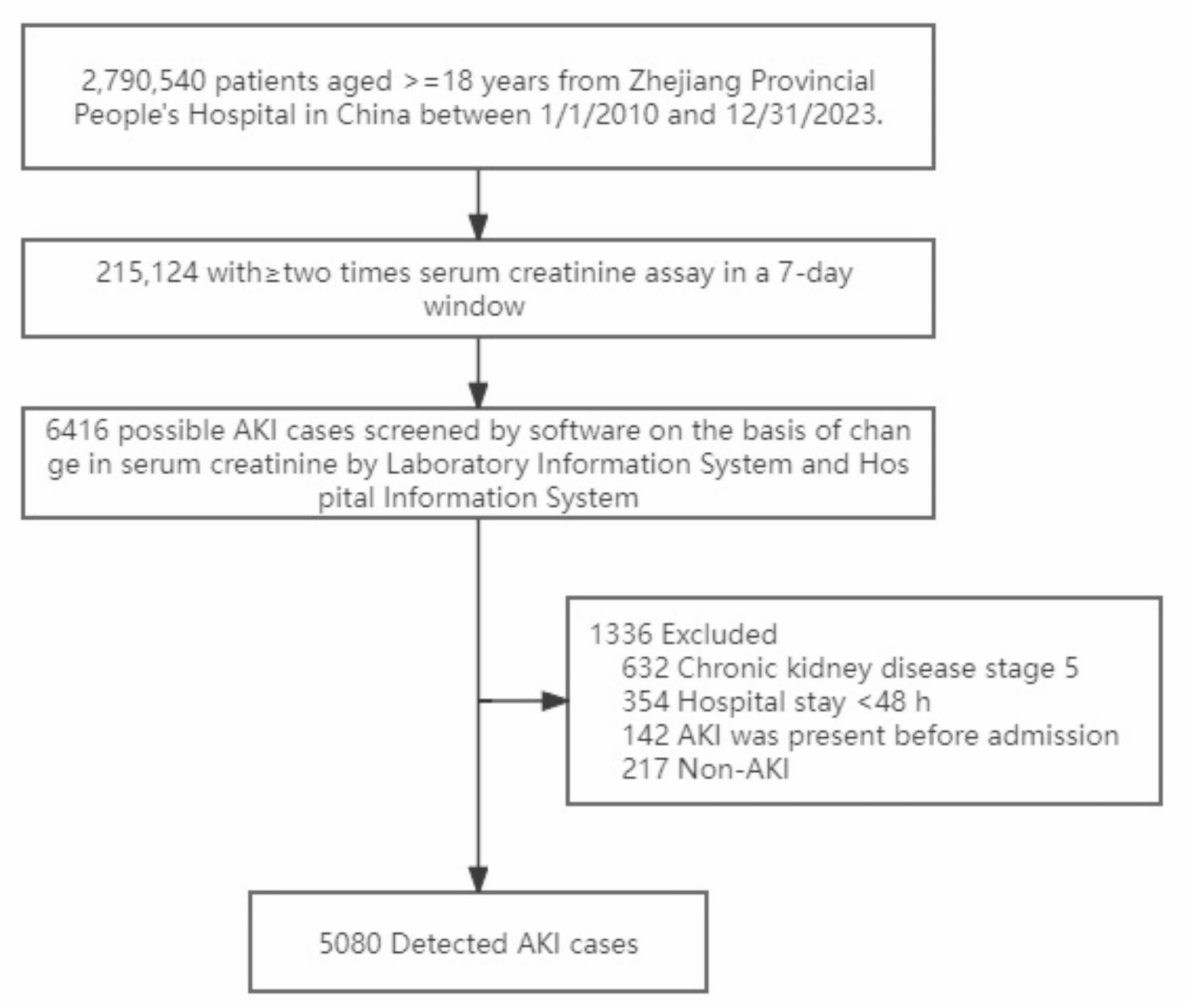
Flow chart of this study.

**Fig. 2:**
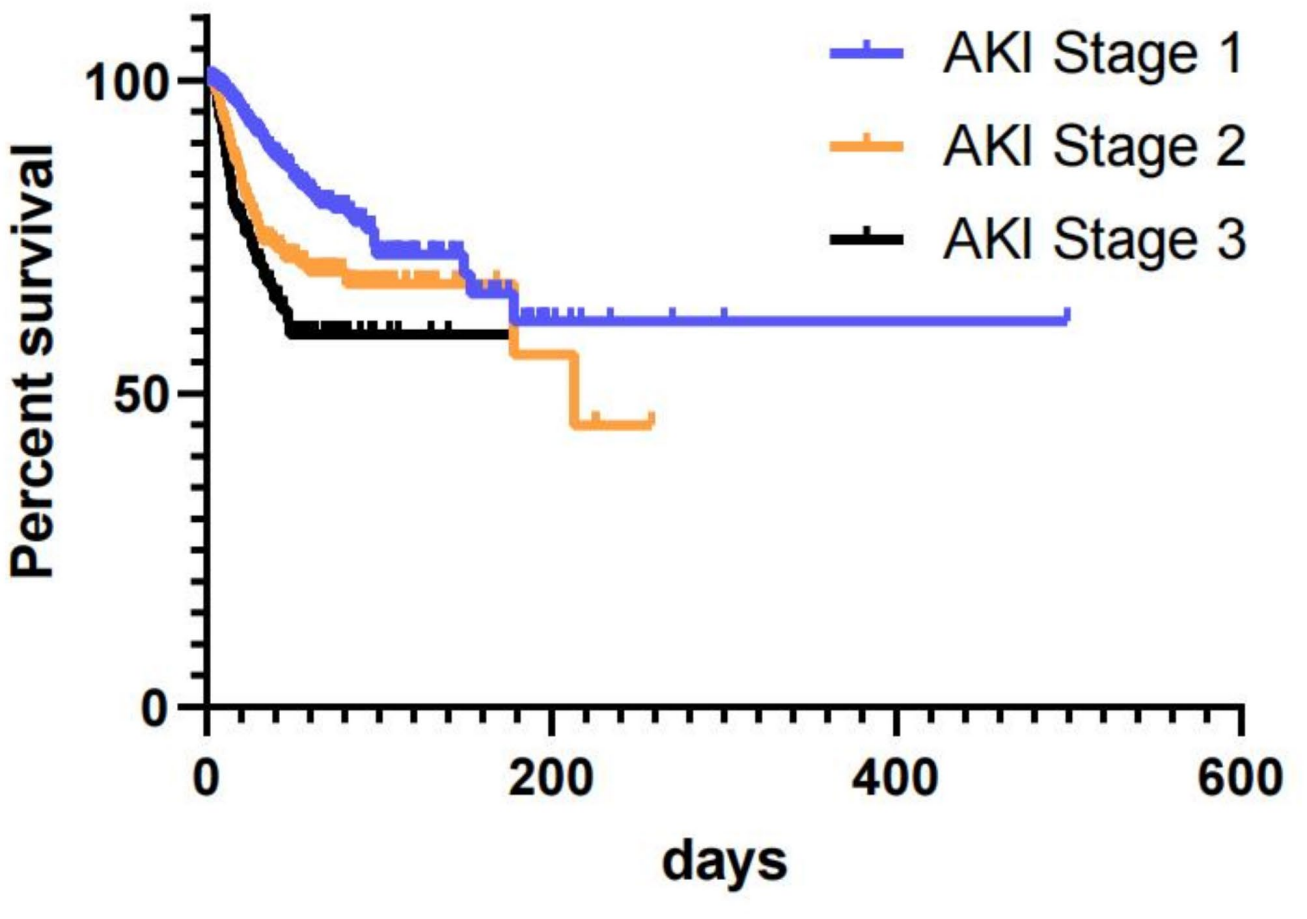
Kaplan–Meier survival curves in AKI stages and the impact on survival days. Log-rank test, *p* < 0.0001.

The records of 2,790,540 patients, including 512,372 hospitalized patients and 2,278,168 patients in the outpatient clinic, were included in this study. Serum creatinine levels were measured at least two or more times in a seven-day period in 215,124 (7.7%) patients. AKI was suspected in 6,416 patients based on changes in serum creatinine levels (Fig. 1). The overall incidence of AKI was 0.18% (5,080/2,790,540), with a hospitalization incidence of 0.78% (4,023/512,372) and an outpatient incidence of 0.05% (1,057/2,278,168). The mean baseline serum creatinine level was 74.5 μmol/L [57.0, 97.8 μmol/L]. The number of patients with stage 1 AKI was 3,422 (67.4%), stage 2 AKI was 1,027 (20.2%), and stage 3 AKI was 631 (12.4%). The average age of patients with AKI at the time of their initial hospital admission was 65.2 years [52.8, 75.8 years]. In the AKI group, the proportion of males was higher than that of females (3,065, 60.3% vs. 2,015, 39.7%), and 43.4% (2,206) of the patients had hypertension, 19.0% (966) had diabetes, 11.8% (597) had heart disease, 20.8% (1,058) had malignant tumors, and 15.6% (792) had shock (Table 1). Death events were observed in 481 patients (9.5%), and an increase in AKI stage was associated with worsened survival prognosis (Fig. 2).

**Table 1:** Characteristics of patients with AKI.

	Overall	Unrecognized AKI	Recognized AKI	*p* value
n	5080	3878	1202	
Outpatient n(%)	877 (17.3)	670 (17.3)	207 (17.2)	0.964
Inpatients n(%)	4203 (82.7)	3208 (82.7)	995 (82.8)	
AKI stage n(%)				
1	3422 (67.4)	2907 (75.0)	515 (42.8)	
2	1027 (20.2)	649 (16.7)	378 (31.4)	< 0.001
3	631 (12.4)	322 (8.3)	309 (25.7)	
Sex n (%)				
Male	3065 (60.3)	2282 (58.8)	783 (65.1)	0.001
Female	2015 (39.7)	1596 (41.2)	419 (34.9)	
Age (year)	65 [53, 76]	65 [52, 75]	67 [56, 78]	0.577
Comorbidity				
Shock n (%)	792 (15.6)	332 (8.6)	460 (38.3)	< 0.001
Heart disease n (%)	597 (11.8)	405 (10.4)	192 (16.0)	< 0.001
Hypertension n (%)	2206 (43.4)	1586 (40.9)	620 (51.6)	< 0.001
Diabetes mellitus n (%)	966 (19.0)	704 (18.2)	262 (21.8)	0.006
Malignant tumor n (%)	1058 (20.8)	851 (21.9)	207 (17.2)	< 0.001
Death n (%)	481 (9.5)	287 (7.4)	194 (16.1)	< 0.001
Smoking habit n (%)	1264 (26.3)	936 (25.8)	328 (27.8)	0.197
Drinking habit n (%)	1025 (21.3)	770 (21.3)	255 (21.6)	0.82
Baseline Scr, umol/L	75 [57, 98]	69 [54, 88]	100 [75, 148]	< 0.001
Max Scr, umol/L	144 [99, 236]	123 [91, 177]	264 [187, 391]	< 0.001
Last Scr, umol/L	101 [72, 162]	90 [68, 133]	166 [111, 261]	< 0.001
Re-check Scr n (%)	3344 (65.8)	2507 (64.6)	837 (69.6)	0.002
AKI recover n (%)				< 0.001
Unrecovered	1012 (19.9)	782 (20.2)	230 (19.1)	
Recovered	1721 (33.9)	1372 (35.4)	349 (29.0)	
Partial recovery	611 (12.0)	353 (9.1)	258 (21.5)	
Missing data	1736 (34.2)	1371 (35.4)	365 (30.4)	
AKI recover time (day)	9.0 [4.0, 17.9]	9.0 [4.9, 17.9]	8.0 [3.0, 17.6]	< 0.001
Follow-up time (day)	15.8 [10.1, 25.4]	15.8 [10.2, 25.2]	15.5 [9.8, 25.9]	0.724
More than 28 days n (%)	1046 (20.6)	781 (20.1)	265 (22.0)	0.165
More than 90 days n (%)	98 (1.9)	70 (1.8)	28 (2.3)	0.301

Scr, serum creatinine; Max Scr, maximal serum creatinine values that occurred during follow-up; Last Scr, last serum creatinine values during follow-up; Re-check Scr, the number of patients whose serum creatinine values were measured again after AKI; More than 28 days: number of patients followed for more than 28 days after AKI onset; More than 90 days: number of patients followed for more than 90 days after AKI onset.

### Unrecognized rate of AKI

In total, 5,080 patients had AKI, including 1,202 with recognized AKI and 3,878 (76.3%) with unrecognized AKI (Table 1). The proportion of outpatients and inpatients was similar between the two groups. Most patients with unrecognized AKI had stage 1 disease (75.0%), followed by stage 2 AKI (16.7%) and stage 3 AKI (8.3%). Patients with unrecognized AKI were less likely to have shock (p < 0.001), heart disease (p < 0.001), hypertension (p < 0.001), and diabetes mellitus (p = 0.006) than those with recognized AKI; however, these results were the opposite in patients with malignant tumors. The baseline serum creatinine and maximum serum creatinine levels were lower in patients with unrecognized AKI than in patients with recognized AKI.

### Trends in AKI unrecognized rates over the study period

**Fig. 3:**
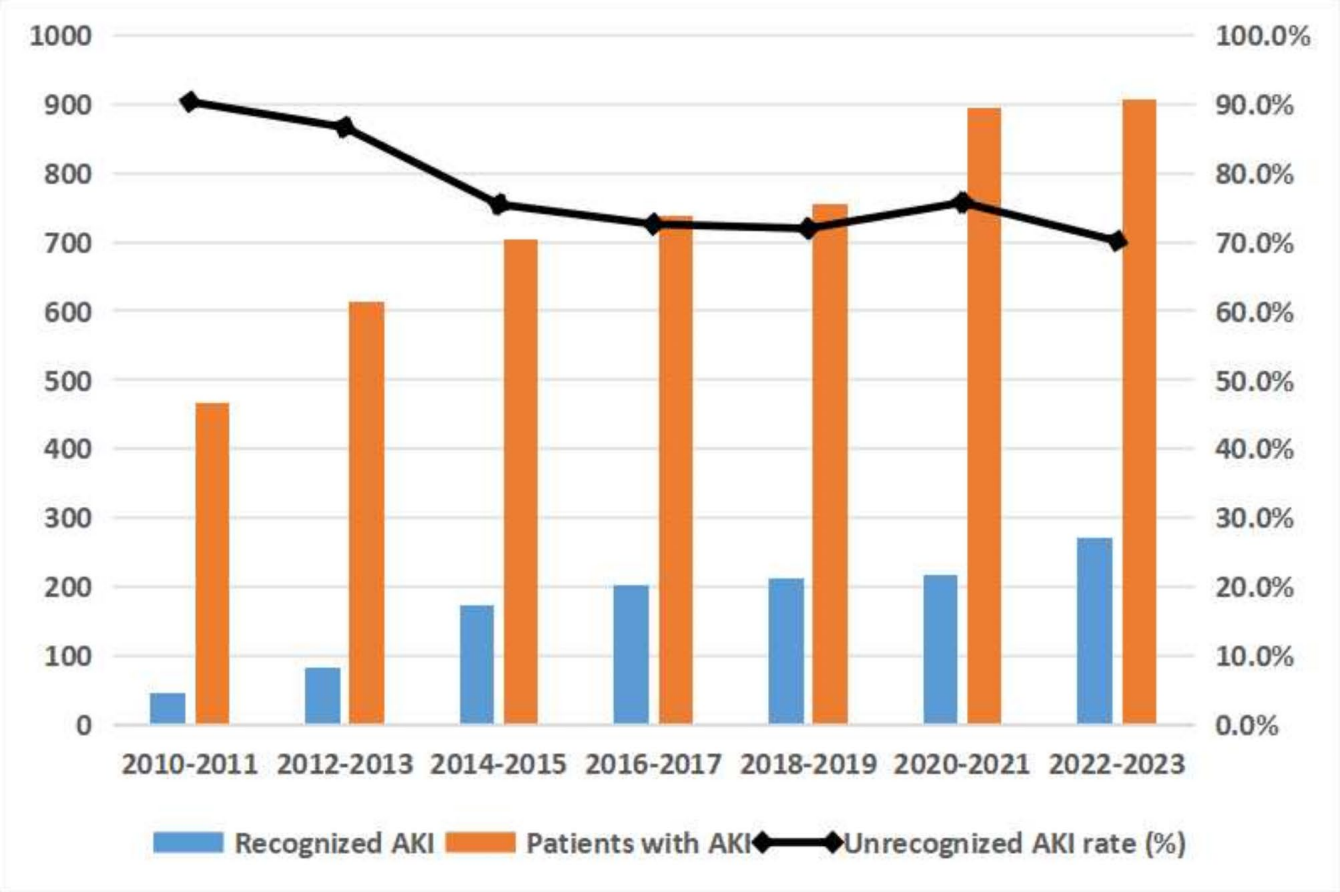
The difference in unrecognized AKI rate in every two-year interval from 2010 to 2023.

**Fig. 4:**
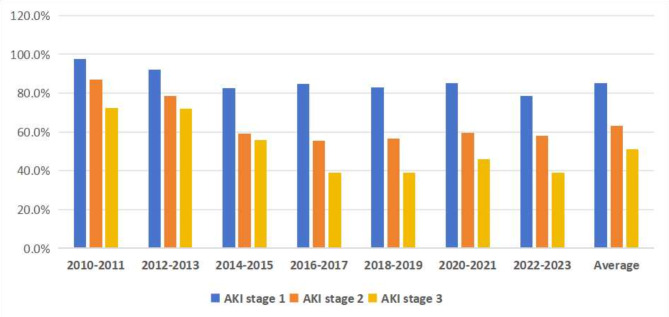
AKI stages in every two-year interval from 2010 to 2023.

Over the 14-year study period, the unrecognized rate of AKI decreased annually from 90.3% in 2010–2011 to 70.2% in 2023–2024. The unrecognized rate of AKI significantly decreased each year since 2014–2015 (Fig. 3 and Supplementary Table S1), when the 2012 KDIGO definition of AKI was officially published. The mean unrecognized rate over the 14-year study period was highest among patients with AKI stage 1 (85.0%), and the annual variation was modest (Fig. 4 and Supplementary Table S2). The average unrecognized rate of AKI stage 3 was the lowest (51.0%) and decreased each year (Fig. 4 and Supplementary Table S2).

### Rate of unrecognized AKI among different hospital departments

**Fig. 5:**
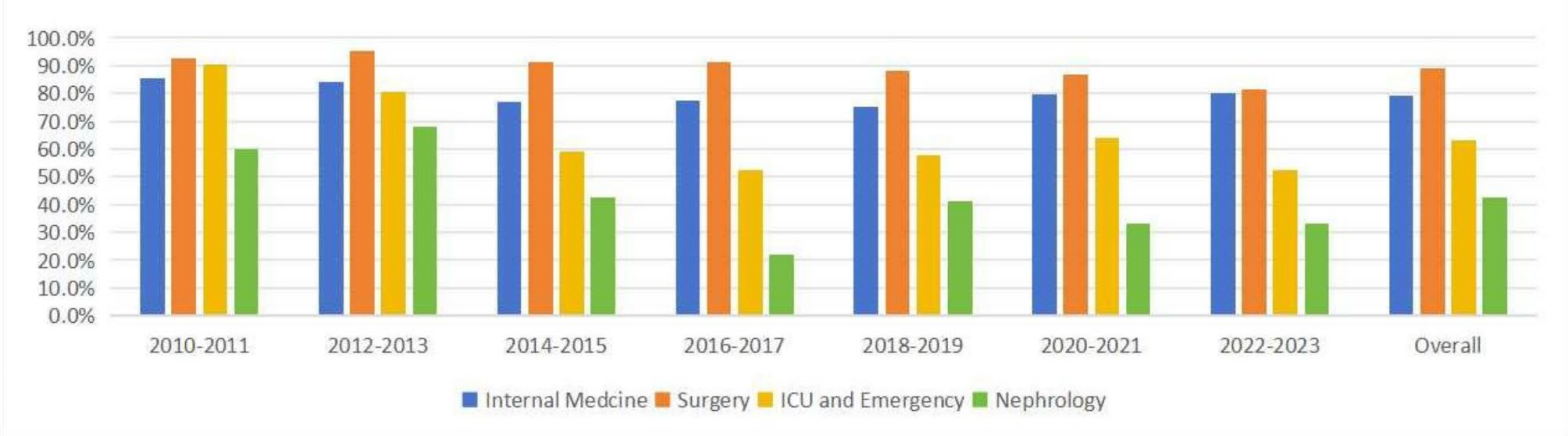
The unrecognized rate of AKI in various departments from 2010 to 2023.

The nephrology department had the lowest missed diagnosis rate of 42.62% (52/122). Among non-nephrology departments, the intensive care unit (55.77%, 532/954) departments had the lowest missed diagnosis rates. The orthopedics department had the highest missed diagnosis rate at 94.48% (137/145) (Table 2). Overall, the nephrology department had the lowest rate of unrecognized AKI over the entire study period (42.62%), followed by the ICU and emergency medicine department (63.30%), internal medicine department (79.20%), and surgical department (89.21%) (Fig. 5 and Supplementary Table S3).

**Table 2:** The unrecognized rate of AKI in different departments.

Department	Recognized AKI	Unrecognized AKI	Overall AKI	Unrecognized AKI rate (%)
Overall	1202	3878	5080	76.3
Cardiology	64	341	405	84.2
Respiratory medicine	23	90	113	79.7
Gastroenterology	11	48	59	81.4
Neurology	42	206	248	83.1
Nephrology	70	52	122	42.6
Oncology	33	104	137	75.9
Hematology department	11	51	62	82.3
Infectious diseases department	48	161	209	77.0
Rehabilitation department	7	99	106	93.4
Other internal medicine	15	82	97	84.5
Overall internal medicine	324	1234	1558	79.2
General Surgery	57	471	528	89.2
Neurosurgery	15	136	151	90.1
Urology	49	397	446	89.0
Thoracic surgery	28	180	208	86.5
Orthopedics	11	137	145	94.5
Other surgical department	13	112	125	89.6
Overall surgical medicine	173	1433	1603	89.4
Emergency	221	577	798	72.3
ICU	422	532	954	55.8
Missing data	62	102	167	61.1

### Low follow-up rate of patient with AKI

The mean follow-up time after AKI was 15.8 days [10.1, 25.4 days]. Patients with recognized and unrecognized AKI had similar follow-up rates at 28 (20.1% vs. 22.0%, respectively, p = 0.165) and 90 (1.8% vs. 2.3%, respectively, p = 0.301) days after AKI (Table 1). The rate of full or partial renal recovery was 50.5% in patients with recognized AKI and 44.5% in patients with unrecognized AKI. The mean AKI recovery time was 9.0 days [4.0–17.9 days] and was faster among patients with recognized AKI than those with unrecognized AKI (8.0 vs. 9.0 days, respectively, *p* < 0.001) (Table 1).

### Risk factors of unrecognized AKI

A multivariate logistic regression model was established with unrecognized AKI as the dependent variable, and lower AKI stage, lower baseline serum creatinine level, absence of shock, absence of heart disease, absence of hypertension, admission to a non-nephrology department, and admission to a surgery department as independent risk factors. Stage 1 AKI (stage 1 vs. stage 3; odds ratio [OR], 4.676; 95% confidence interval [CI], 3.787–5.775; p < 0.001), stage 2 AKI (stage 2 vs. stage 3; OR, 1.81; 95% CI, 1.436–2.281; p < 0.001), baseline serum creatinine (OR, 0.983; 95% CI, 0.981–0.985; p < 0.001), shock (OR, 0.196; 95% CI, 0.163–0.237; p < 0.001), heart disease (OR, 0.717; 95% CI, 0.570–0.902; p = 0.004), hypertension (OR, 0.696; 95% CI, 0.587–0.825; p < 0.001), admission to the surgery department (OR, 2.223; 95% CI, 1.812–2.728; p < 0.001), and admission to a non–nephrology department (OR, 0.346; 95% CI, 0.218–0.549; p < 0.001) were identified as independent variables (Table 3). The tolerance was > 0.85, and the VIF was < 10, indicating no collinearity among the independent variables (Supplementary Table S4).

**Table 3:** Multivariable logistic regression analysis of unrecognized AKI.

Variables	OR	Odds ratio (95% CI)	*P* value
Age	0.996	0.991–1.002	0.167
Sex (male)	0.958	0.812–1.131	0.616
AKI			
Stage 3	Reference	Reference	
Stage 1	4.676	3.787–5.775	< 0.001
Stage 2	1.810	1.436–2.281	< 0.001
Baseline serum creatinine	0.983	0.981–0.985	< 0.001
Shock	0.196	0.163–0.237	< 0.001
Heart disease	0.717	0.570–0.902	0.004
Hypertension	0.696	0.587–0.825	< 0.001
Diabetes mellitus	0.974	0.796–1.190	0.795
Malignant tumor	0.961	0.780–1.183	0.705
Surgery department	2.223	1.812–2.728	< 0.001
Nephrology department	0.346	0.218–0.549	< 0.001

## Discussion

AKI is characterized by a rapid decline in kidney function and potential multi-organ failure, leading to increased hospitalization time, medical costs, and mortality. Early recognition and intervention are crucial for the management of AKI. However, current identification rates remain low, especially outside nephrology departments, highlighting the necessity of this research.

This study used a large-scale, retrospective cohort design with electronic medical records to investigate AKI incidence, unrecognized rate, and follow-up outcomes. The overall AKI incidence was 0.18%, with higher rates in hospitalized patients than outpatients. The hospitalized AKI incidence is comparable to a previous study^12,13^. Few studies have analyzed outpatient AKI due to the large volume of data and lack of robust analysis platforms^14^. About three-fourths of AKI cases were unrecognized, mostly AKI stage 1. Unrecognition rates varied across departments, with the lowest in the ICU and the highest in orthopedics. The high rate of unrecognized AKI and its association with poor outcomes, especially in non-nephrology departments, underscores the need for improved diagnostic protocols. This finding is consistent with Chertow et al.^15^. The unrecognized rate gradually decreased over the study period. Low follow-up rate and short follow-up time of patient with AKI was presented. Lower AKI stage, lower baseline serum creatinine, absence of shock, heart disease, or hypertension, and admission to non-nephrology or surgery departments were identified as independent risk factors for unrecognized AKI, which providing a deeper scientific investigation of this clinical issue. These findings highlight the need for enhanced AKI monitoring and early intervention to improve patient outcomes and reduce healthcare costs.

The tertiary academic hospital included in this study is located in a prosperous region of China. From 2016–2017, the rate of unrecognized AKI at this center (Supplementary Table S1) was similar to that in other academic hospitals in the same period^4^. Although the rate of unrecognized early-stage AKI decreased significantly from 2014–2015 after the official publication of the 2012 KDIGO AKI definition in 2013, it remained high in 2022–2023. This may be due to several factors: First, physicians in China, especially non-nephrologists, lack knowledge about diagnosing, treating, and preventing AKI. This results in inadequate renal function monitoring and high rates of missed AKI diagnoses, leading to poor patient outcomes^16^. Second, despite the publication of a consensus definition of AKI over a decade ago, inconsistent application of guidelines has led to low awareness among physicians. There are over 60 definitions of AKI, with no unified diagnostic standard, causing inconsistent diagnostic proficiency^17,18^. Third, AKI often occurs without obvious symptoms, particularly in stage 1 AKI, which has the highest unrecognized rate due to milder symptoms. This poses a challenge for non-nephrologists. Additionally, even when identified by other specialists, patients with AKI are rarely referred to nephrologists. In Glasgow, 23.5% of 1,500 patients had unrecognized AKI, and two-thirds were discharged without resolution^19^. In the UK, only 22–31% of AKI patients are referred to nephrologists, with a bias against older patients and those with more comorbidities^13^.

A large cross-sectional study found that over 50% of ICU patients experience AKI, with an overall incidence of 32.9%^20^. In this study, while the ICU and emergency department had the highest AKI diagnosis rates, more than 50% of AKI cases went unrecognized, highlighting the need for better training and diagnostic tools. Among 376 AKI patients, 37.2% (140/376) were undiagnosed, leading to poorer outcomes^21^. A study at China’s Huoshenshan Hospital showed that undiagnosed AKI patients with COVID-19 had shorter hospital stays and higher mortality rates compared to diagnosed patients^22^. Unrecognized AKI results in longer recovery times and worse survival outcomes. These findings suggest the need for standardized AKI monitoring across hospital departments to improve patient outcomes and reduce healthcare costs. Routine AKI screening and machine-learning algorithms could enhance early detection.

Timely recognition of AKI is crucial. This study highlights that unrecognized AKI leads to longer recovery times, higher medical expenses, and poorer patient outcomes. The longer the recovery time, the higher the medical expenses and the poorer the patient prognosis^23^. The UK National Confidential Enquiry into Patient Outcome and Death recommends including AKI risk assessments for all emergency department admissions^24^. Identifying AKI helps determine the cause of kidney damage. Although the overall mortality rate is low (9.5% in this study), AKI significantly increases the risk of mortality compared to previous meta-analyses^24^. Early intervention by nephrologists can help patients recover and prevent further kidney damage. Nephrologist involvement is linked to a lower risk of starting renal replacement therapy and AKI progression^25,26^. To improve AKI recognition, healthcare workers need training, and nephrology specialists should educate on renal disease. For patients suspected of having AKI, kidney function tests should be more frequent. Monitoring and recording urine output is also critical for patients with abnormal kidney function.

In this study, the follow-up time post-discharge was short, and both groups had low 90-day follow-up rates. This could be due to missed AKI diagnoses, leading to insufficient health education and patient awareness. Additionally, the referral system needs improvement, as patients often opt for large hospitals, and information sharing between hospitals is still in its early stages. Follow-up during the kidney recovery phase is crucial. Forni et al. recommend that all AKI patients, regardless of renal recovery, should be monitored for CKD development^27^. A pop-up reminder in the outpatient system for AKI patients would help doctors remember to review kidney function. Embedding an AKI early warning system into the electronic medical record system can dynamically predict the risk of AKI in patients in real time, helping clinicians to implement precise prevention and control measures^28^. Collaboration with patient support systems and government assistance in integrating medical data are also essential to improve follow-up rates.

This study has several limitations. First, the retrospective design may introduce information bias as the data were collected from existing electronic medical records, which may not capture all relevant clinical details. Second, the study was conducted at a single institution, which may limit the generalizability of the findings to other settings or populations. Third, the study did not analyze the impact of different treatment modalities on the prognosis of patients with AKI, which may provide more comprehensive insights into the optimization of patient outcomes. Fourth, We did not account for whether patients were readmitted and developed a second AKI. Consequently, there may be a risk of underestimating the total number of AKI cases.These limitations suggest that future research should include prospective multicenter studies and a detailed examination of treatment strategies to validate and extend these findings.

In conclusion, the high rate of unrecognized AKI and its association with poorer prognosis and longer recovery times underscore the urgent need for improved early detection and intervention strategies. The lack of familiarity with the guidelines for diagnosing AKI among non-nephrology physicians may lead to a low awareness of the diagnosis of the disease. Strategies should be implemented to improve early recognition and follow-up of patients with AKI, especially in high-risk groups. The independent risk factors identified in this study are expected to serve as targets for future interventions. The establishment of an early warning system for AKI in the electronic medical record system may help solve the problem of a high rate of unrecognized AKI.

## Electronic supplementary material

Below is the link to the electronic supplementary material.

Supplementary Material 1

Supplementary Material 2

Supplementary Material 3

Supplementary Material 4

## Data Availability

The datasets of this study are available from the corresponding author on reasonable request.

## References

[1] Kellum, J. et al. Kidney disease: Improving global outcomes (KDIGO) acute kidney injury work group. KDIGO clinical practice guideline for acute kidney injury. *Kidney Int. Suppl.* **2**, 1–138 (2012).

[2] Cheng, X., Wu, B., Liu, Y., Mao, H. & Xing, C. Incidence and diagnosis of acute kidney injury in hospitalized adult patients: A retrospective observational study in a tertiary teaching Hospital in Southeast China. *BMC Nephrol.* **18**, 203. 10.1186/s12882-017-0622-6 (2017). 28646870 10.1186/s12882-017-0622-6 PMC5483286

[3] Yan, P. et al. Acute kidney disease in hospitalized acute kidney injury patients. *PeerJ* **9**, e11400. 10.7717/peerj.11400 (2021). 34113486 10.7717/peerj.11400 PMC8158174

[4] Yang, L. et al. Acute kidney injury in China: A cross-sectional survey. *Lancet* **386**, 1465–1471. 10.1016/S0140-6736(15)00344-X (2015). 26466051 10.1016/S0140-6736(15)00344-X

[5] Kathleen, D. L. et al. AKI!Now initiative: recommendations for awareness, recognition, and management of AKI. *Clin. J. Am. Soc. Nephrol.* **15**(12), 1838–1847. 10.2215/CJN.15611219 (2020). 32317329 10.2215/CJN.15611219 PMC7769012

[6] Patsalis, N. et al. Early risk predictors of acute kidney injury and short-term survival during Impella support in cardiogenic shock. *Sci. Rep.* **14**(1), 17484. 10.1038/s41598-024-68376-w (2024). 39080441 10.1038/s41598-024-68376-w PMC11289486

[7] Forni, L. G. et al. Renal recovery after acute kidney injury. *Intensive Care Med.* **43**, 855–866. 10.1007/s00134-017-4809-x (2017). 28466146 10.1007/s00134-017-4809-x PMC5487594

[8] Wald, R. et al. The association between renal replacement therapy modality and long-term outcomes among critically ill adults with acute kidney injury: A retrospective cohort study. *Crit. Care Med.* **42**, 868–877. 10.1097/CCM.0000000000000042 (2014). 24275513 10.1097/CCM.0000000000000042

[9] Li, Q. et al. Missed diagnosis of acute kidney injury in older patients with invasive mechanical ventilation: A multicenter retrospective study. *Aging Clin. Exp. Res.* **34**, 2887–2895. 10.1007/s40520-022-02229-2 (2022). 36029419 10.1007/s40520-022-02229-2

[10] Liu, W. et al. Hospital-acquired acute kidney injury in older patients: Clinical characteristics and drug analysis. *Gerontology* **68**, 763–770. 10.1159/000518938 (2022). 34537763 10.1159/000518938

[11] Guthrie, G. et al. Developing an AKI consensus definition for database research: Findings from a scoping review and expert opinion using a Delphi process. *Am. J. Kidney Dis.* **79**, 488-496.e1. 10.1053/j.ajkd.2021.05.019 (2022). 34298142 10.1053/j.ajkd.2021.05.019

[12] Ping, C., Yun, Z., Min, M., Rongshan, L. I. & Tao, W. Analysis of missed diagnosis of acute kidney injury in non-nephrological hospitalized adult patients. *Chin. J. Nephrol.* **30**, 645–649 (2014).

[13] Hsu, C. Y. et al. Community-based incidence of acute renal failure. *Kidney Int.* **72**, 208–212. 10.1038/sj.ki.5002297 (2007). 17507907 10.1038/sj.ki.5002297 PMC2673495

[14] Lydia, A. Raising awareness of acute kidney injury: Unfolding the truth. *Acta Med. Indones.* **54**, 513–516 (2022). 36624719

[15] Chertow, G. M., Burdick, E., Honour, M., Bonventre, J. V. & Bates, D. W. Acute kidney injury, mortality, length of stay, and costs in hospitalized patients. *J. Am. Soc. Nephrol.* **16**, 3365–3370. 10.1681/ASN.2004090740 (2005). 16177006 10.1681/ASN.2004090740

[16] Khatana, J., Thavamani, A., Umapathi, K. K., Sankararaman, S. & Roy, A. Increasing incidence of acute kidney injury in pediatric severe sepsis and related adverse hospital outcomes. *Pediatr. Nephrol.* **38**, 2809–2815. 10.1007/s00467-022-05866-x (2023). 36622440 10.1007/s00467-022-05866-x

[17] Puzanov, A., Tkachuk, V. & Maksymenko, A. Acute kidney injury after arterial switch operation: Incidence, risk factors, clinical impact – A retrospective single-center study. *Ren. Fail.* **45**, 2167661. 10.1080/0886022X.2023.2167661 (2023). 36692196 10.1080/0886022X.2023.2167661 PMC9879166

[18] Pande, S. D. et al. Acute kidney injury without need for dialysis, incidence, its impact on long-term stroke survival and progression to chronic kidney disease. *BMJ Open* **12**, e050743. 10.1136/bmjopen-2021-050743 (2022). 35613807 10.1136/bmjopen-2021-050743 PMC9134210

[19] German EHEC-HUS Registry. The German 2011 epidemic of Shiga toxin-producing E. Coli-the nephrological view. *Nephrol. Dial. Transplant.* **26**(9), 2723–2726. 10.1093/ndt/gfr462 (2011). 21852273 10.1093/ndt/gfr462

[20] Santos, P. R. & Monteiro, D. L. Acute kidney injury in an intensive care unit of a general hospital with emergency room specializing in trauma: an observational prospective study. *BMC Nephrol.* **16**, 30. 10.1186/s12882-015-0026-4 (2015). 25885883 10.1186/s12882-015-0026-4 PMC4377071

[21] White, K. C. et al. Sepsis-associated acute kidney injury in the intensive care unit: Incidence, patient characteristics, timing, trajectory, treatment, and associated outcomes. A multicenter, observational study. *Intensive Care Med.* **49**, 1079–1089. 10.1007/s00134-023-07138-0 (2023). 37432520 10.1007/s00134-023-07138-0 PMC10499944

[22] Li, Q., Hu, P., Kang, H. & Zhou, F. Clinical characteristics and short-term outcomes of acute kidney injury missed diagnosis in older patients with severe COVID-19 in Intensive care unit. *J. Nutr. Health Aging* **25**, 492–500. 10.1007/s12603-020-1550-x (2021). 33786567 10.1007/s12603-020-1550-x PMC7754698

[23] Nateghi Haredasht, F. et al. The effect of different consensus definitions on diagnosing acute kidney injury events and their association with in-hospital mortality. *J. Nephrol.* **35**, 2087–2095. 10.1007/s40620-022-01323-y (2022). 35441981 10.1007/s40620-022-01323-y

[24] Liu, K. D. et al. AKI!Now initiative: Recommendations for awareness, recognition, and management of AKI. *Clin. J. Am. Soc. Nephrol.* **15**, 1838–1847. 10.2215/CJN.15611219 (2020). 32317329 10.2215/CJN.15611219 PMC7769012

[25] Lewington, A. J., Cerdá, J. & Mehta, R. L. Raising awareness of acute kidney injury: A global perspective of a silent killer. *Kidney Int.* **84**, 457–467. 10.1038/ki.2013.153 (2013). 23636171 10.1038/ki.2013.153 PMC3758780

[26] Chávez-Íñiguez, J. S. et al. Nephrologist interventions to avoid kidney replacement therapy in acute kidney injury. *Kidney Blood Press. Res.* **46**, 629–638. 10.1159/000517615 (2021). 34315155 10.1159/000517615

[27] Chawla, L. S. et al. Acute kidney disease and renal recovery: Consensus report of the acute disease quality initiative (ADQI) 16 workgroup. *Nat. Rev. Nephrol.* **13**, 241–257. 10.1038/nrneph.2017.2 (2017). 28239173 10.1038/nrneph.2017.2

[28] Zhang, Y. et al. Development and validation of a real-time prediction model for acute kidney injury in hospitalized patients. *Nat. Commun.* **16**, 68. 10.24433/CO.9261428.v1 (2025). 39747882 10.1038/s41467-024-55629-5 PMC11695981

