# Supplementary material for: Unexpectedly high rate of unrecognized acute kidney injury and its trend over the past 14 years
Source: Sci Rep. 2025 Feb 21;15:6305. doi: 10.1038/s41598-025-88732-8 (PMC11845613; doi:10.1038/s41598-025-88732-8)
Supplement: Supplementary file 1 — Supplementary Material 1 [file 41598_2025_88732_MOESM1_ESM.docx]

| Supplement Table 1: The difference of unrecognized AKI rate in every two-year interval | | | | | | | |
| --- | --- | --- | --- | --- | --- | --- | --- |
|  | 2010-2011 | 2012-2013 | 2014-2015 | 2016-2017 | 2018-2019 | 2020-2021 | 2022-2023 |
| Recognized AKI | 45 | 82 | 173 | 202 | 212 | 217 | 271 |
| Admissions | 466 | 614 | 705 | 737 | 756 | 894 | 908 |
| Unrecognized AKI rate (%) | 90.3% | 86.6% | 75.5% | 72.6% | 72.0% | 75.7% | 70.2% |
