# Supplementary material for: Unexpectedly high rate of unrecognized acute kidney injury and its trend over the past 14 years
Source: Sci Rep. 2025 Feb 21;15:6305. doi: 10.1038/s41598-025-88732-8 (PMC11845613; doi:10.1038/s41598-025-88732-8)
Supplement: Supplementary file 2 — Supplementary Material 2 [file 41598_2025_88732_MOESM2_ESM.docx]

| Supplement Table 2: Unrecognized AKI rate in differ stages every two-year interval | | | |
| --- | --- | --- | --- |
| Year | AKI stage 1 | AKI stage 2 | AKI stage 3 |
| 2010-2011 | 97.4% | 87.0% | 72.4% |
| 2012-2013 | 92.1% | 78.5% | 72.1% |
| 2014-2015 | 82.5% | 59.0% | 55.9% |
| 2016-2017 | 84.8% | 55.3% | 38.8% |
| 2018-2019 | 82.8% | 56.6% | 39.1% |
| 2020-2021 | 85.2% | 59.6% | 45.8% |
| 2022-2023 | 78.5% | 57.9% | 38.8% |
| Average | 85.0% | 63.2% | 51.0% |
