# Supplementary material for: Unexpectedly high rate of unrecognized acute kidney injury and its trend over the past 14 years
Source: Sci Rep. 2025 Feb 21;15:6305. doi: 10.1038/s41598-025-88732-8 (PMC11845613; doi:10.1038/s41598-025-88732-8)
Supplement: Supplementary file 3 — Supplementary Material 3 [file 41598_2025_88732_MOESM3_ESM.docx]

| Supplement Table 3:: The unrecognized rate of AKI in various departments from 2010 to 2023. | | | | |
| --- | --- | --- | --- | --- |
| Years | Internal Medicine | Surgery | ICU and Emergency | Nephrology |
| 2010-2011 | 85.56% | 92.40% | 90.53% | 60.00% |
| 2012-2013 | 84.18% | 95.24% | 80.57% | 68.18% |
| 2014-2015 | 77.03% | 91.24% | 59.09% | 42.31% |
| 2016-2017 | 77.35% | 91.29% | 52.17% | 21.74% |
| 2018-2019 | 75.32% | 88.16% | 57.82% | 41.18% |
| 2020-2021 | 79.46% | 86.80% | 64.16% | 33.33% |
| 2022-2023 | 80.20% | 81.51% | 52.21% | 33.33% |
| Average | 79.20% | 89.21% | 63.30% | 42.62% |
