# Supplementary material for: Unexpectedly high rate of unrecognized acute kidney injury and its trend over the past 14 years
Source: Sci Rep. 2025 Feb 21;15:6305. doi: 10.1038/s41598-025-88732-8 (PMC11845613; doi:10.1038/s41598-025-88732-8)
Supplement: Supplementary file 4 — Supplementary Material 4 [file 41598_2025_88732_MOESM4_ESM.docx]

| Supplement Table 4: The tolerance and VIF for the dataset | | |
| --- | --- | --- |
| Variables | Tolerance | VIF |
| Age | 0.877 | 1.140 |
| Sex | 0.956 | 1.047 |
| AKI | 0.934 | 1.071 |
| Baseline serum creatinine | 0.905 | 1.106 |
| Shock | 0.911 | 1.098 |
| Heart disease | 0.939 | 1.065 |
| Hypertension | 0.850 | 1.176 |
| Diabetes mellitus | 0.925 | 1.081 |
| Malignant tumor | 0.911 | 1.098 |
| Surgery department | 0.867 | 1.154 |
| Nephrology department | 0.937 | 1.068 |

VIF, variance inflation factor; AKI: acute kidney injury.
